# Impairment of the Hypothalamus–Pituitary–Thyroid Axis Caused by Naturally Occurring *GATA2* Mutations In Vitro

**DOI:** 10.3390/ijms221810015

**Published:** 2021-09-16

**Authors:** Yuki Sakai, Kenji Ohba, Shigekazu Sasaki, Akio Matsushita, Hiroko Misawa Nakamura, Go Kuroda, Daisuke Tsuriya, Miho Yamashita, Takafumi Suda

**Affiliations:** 1 Second Division, Department of Internal Medicine, Hamamatsu University School of Medicine, Shizuoka 431-3192, Japan; ysakai@hama-med.ac.jp (Y.S.); akiom@hama-med.ac.jp (A.M.); hironee@hama-med.ac.jp (H.M.N.); gokuroda@hama-med.ac.jp (G.K.); dtsuri@hama-med.ac.jp (D.T.); mihojy@hama-med.ac.jp (M.Y.); suda@hama-med.ac.jp (T.S.); 2 Medical Education Center, Hamamatsu University School of Medicine, Shizuoka 431-3192, Japan; 3 International Center, Hamamatsu University School of Medicine, Shizuoka 431-3192, Japan

**Keywords:** GATA, thyroid, central hypothyroidism, hypothalamus–pituitary–thyroid axis (HPT axis), haploinsufficiency

## Abstract

The transcription factor GATA2 regulates gene expression in several cells and tissues, including hematopoietic tissues and the central nervous system. Recent studies revealed that loss-of-function mutations in *GATA2* are associated with hematological disorders. Our earlier in vitro studies showed that GATA2 plays an essential role in the hypothalamus–pituitary–thyroid axis (HPT axis) by regulating the genes encoding prepro-thyrotropin-releasing hormone (preproTRH) and thyroid-stimulating hormone β (TSHβ). However, the effect of GATA2 mutants on the transcriptional activity of their promoters remains unelucidated. In this study, we created five human *GATA2* mutations (*R308P*, *T354M*, *R396Q*, *R398W*, and *S447R*) that were reported to be associated with hematological disorders and analyzed their functional properties, including transactivation potential and DNA-binding capacity toward the *preproTRH* and the *TSHβ* promoters. Three mutations (*T354M*, *R396Q*, and *R398W*) within the C-terminal zinc-finger domain reduced the basal GATA2 transcriptional activity on both the *preproTRH* and the *TSHβ* promoters with a significant loss of DNA binding affinity. Interestingly, only the *R398W* mutation reduced the GATA2 protein expression. Subsequent analysis demonstrated that the *R398W* mutation possibly facilitated the GATA2 degradation process. R308P and S447R mutants exhibited decreased transcriptional activity under protein kinase C compared to the wild-type protein. In conclusion, we demonstrated that naturally occurring *GATA2* mutations impair the HPT axis through differential functional mechanisms in vitro.

## 1. Introduction

The hypothalamus–pituitary–thyroid axis (HPT axis) regulates a wide range of processes, including development, growth, and cellular metabolism, with thyroid hormone availability [1,2]. Briefly, thyrotropin-releasing hormone (TRH) stimulates the anterior pituitary to synthesize and release thyrotropin (thyroid-stimulating hormone; TSH), which then acts on the thyroid gland to synthesize and secrete thyroid hormones, including the active form triiodothyronine (T_3_) and the prohormone thyroxine. The effects of thyroid hormones are mainly mediated by the transcriptional activity of thyroid hormone receptors (TRs). TRs are encoded by the genes *TRα* and *TRβ* [3]. *TRα1* and *TRα2* mRNAs are generated via alternative splicing of *TRα*, whereas the use of different promoters in TRβ generates *TRβ1* and *TRβ2* mRNAs. TRH is encoded by the *prepro-thyrotropin-releasing hormone* (*preproTRH)* gene, which generates mature TRH through multiple processing steps [2]. TSH is a glycoprotein hormone comprising a heterodimer with an α (chorionic gonadotropin α) and a β subunit (TSHβ). The latter is unique and confers specificity to TSH action.

In 1997, Gordon et al. demonstrated that transcriptional factors Pit1 and GATA2 are co-expressed in pituitary thyrotropes and stimulate transcription of *TSHβ* [4]. In vivo analysis subsequently showed that co-expression of GATA2 with Pit1 determines thyrotroph differentiation in the pituitary [5]. Charles et al. reported that mice for pituitary-specific inactivation of *Gata2* had fewer thyrotropic cells at birth and that adult mutants produced less TSH in response to severe hypothyroidism after radiothyroidectomy [6]. Our previous in vitro study revealed that the main transcriptional activator of *TSHβ* is GATA2 and not Pit1 [7].

Liu et al. revealed that the expressions of *GATA2* and *TRβ2* are induced when the transcription factors SIM and ARNT2 are stably expressed in the neuronal cell line Neuro-2a [8]. Notably, these transcription factors are essential for the differentiation of the paraventricular nucleus in the hypothalamus [9]. Our recent studies further revealed that GATA2 is a critical activator of the *preproTRH* gene [10].

The GATA family comprises six zinc-finger transcription factors that recognize the consensus DNA motif (A/T) GATA (A/G) in the promoter region of target genes [11]. The hematopoietic GATA factors GATA1, GATA2, and GATA3 are primarily expressed in hematopoietic cells, whereas the nonhematopoietic GATA factors GATA4, GATA5, and GATA6 are expressed in cardiac, hepatic, and intestinal tissues [12]. Regarding GATA2, the expression has been reported in several cells or tissues, including not only hematopoietic tissues but also the central nervous system [13].

Recent experimental and clinical evidence revealed that the loss-of-function mutations of these GATAs are associated with a wide range of disorders, such as congenital heart diseases or hypoparathyroidism, sensorineural deafness, and renal dysplasia (HDR) syndrome [12,14]. GATA2 plays an essential role in the proliferation and the differentiation of hematopoietic cells [15]. In 2011, germline heterozygous mutations in *GATA2* were identified to cause four previously described hematological syndromes: MonoMAC syndrome, dendritic cell, monocyte, and lymphoid deficiency (DCML), familial myelodysplastic/acute myeloid leukemia syndromes, and Emberger syndrome [16]. Although GATA2 plays an essential role in regulating both the *preproTRH* and the *TSHβ* genes [7,10,17,18], little is known about the effect of naturally occuring *GATA2* mutations on the HPT axis.

It was reported that some patients with GATA2 deficiency have not only hematological disorders but also clinical features, such as sensorineural hearing loss, miscarriage, and hypothyroidism [19,20]. Spinner et al. reviewed the medical records of patients with *GATA2* mutations, wherein 8 of 57 (14%) patients had idiopathic hypothyroidism requiring thyroid hormone replacement [19]. Importantly, the prevalence of hypothyroidism reported in their study was much higher than that in the general population, ranging from 0.3% to 0.4% [21]. Unfortunately, because information about whether hypothyroidism is primary or central is lacking in the literature, *GATA2* mutations may predispose patients to develop primary hypothyroidism caused by Hashimoto’s thyroiditis. Although further studies are required to evaluate the clinical characteristics of hypothyroidism by measuring serum free thyroxine, T_3_, and TSH concentrations in the presence or the absence of TRH administration in patients with *GATA2* mutations, it is significant to validate the possible association between GATA2 deficiency and the HPT axis using in vitro models.

In this study, we created five human GATA2 mutants that were reported to be associated with hematological disorders [22,23,24,25,26,27,28]. Additionally, we analyzed their functional properties on the *preproTRH* and the *TSHβ* promoters, including the transactivation potential and the DNA binding capacity. Our findings suggest that *GATA2* mutations impair the HPT axis through differential functional mechanisms in vitro.

## 2. Results

### 2.1. Characteristics of GATA2 Mutations Studied

**Figure 1:**
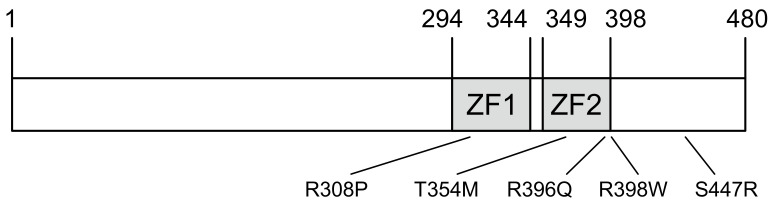
Characteristics of the *GATA2* mutations in this study. The domain of human GATA2 protein showing N- and C-terminal zinc fingers (ZF1, ZF2).

GATA2 contains two highly conserved zinc-finger domains (ZFs) responsible for its DNA binding ability and interaction with other proteins (Figure 1). To investigate the effect of human *GATA2* mutations on the HPT axis, we first selected T354M, R396Q, and R398W, because the major mutational types reported include missense mutations within the C-terminal zinc finger domain (ZF2) [16,22,25,27]. Missense mutations within the N-terminal zinc-finger domain (ZF1) or the C-terminal domain were also reported in patients with GATA2 haploinsufficiency associated with hematological disorders [23,24,26,28]. Therefore, we created two additional mutations, namely R308P within ZF1 and S447R within the C-terminal domain, to compare them with three mutants.

### 2.2. The GATA2 Mutants Have Reduced Transcriptional Activity on the preproTRH Promoter

**Figure 2:**
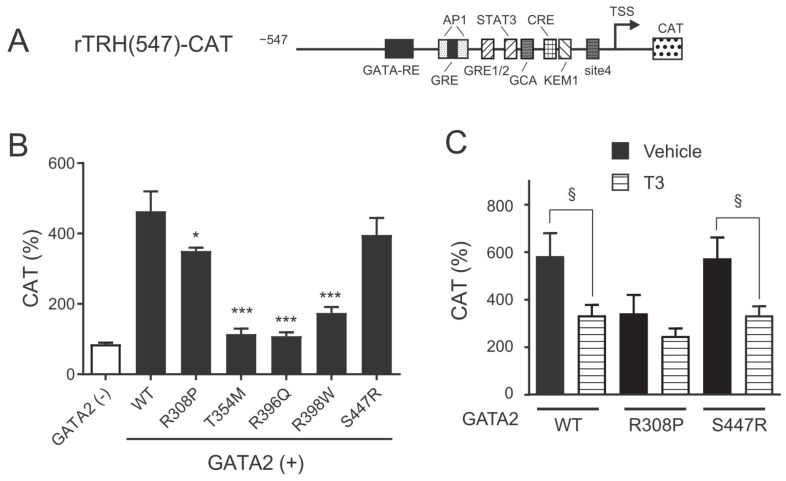
*GATA2* mutations impair basal *preproTRH* transcriptional activity. (**A**) Schematic representation of rTRH(547)-CAT. GATA-RE; GATA responsive element; AP-1, AP-1-binding site; GRE, glucocorticoid responsive element; GRE1/2, glucocorticoid responsive element half-site; STAT, signal transducer and activator of transcription; CRE, cyclic AMP response element; GCA, GCA site; KEM1, KEM1 site; TSS, transcription start site; CAT, chloramphenicol acetyltransferase. (**B**) and (**C**) rTRH(547)-CAT (1.0 μg) was transfected into CV1 cells along with expression plasmids for various GATA2 mutants (0.1 μg), TRβ2 (0.1 μg), and pCMV-β-galactosidase (0.2 μg). After 24 h of culture, the cells were treated with vehicle or 10 nM T_3_ for an additional 24 h. CAT activity was normalized with β-galactosidase activity. CAT activity for pCMV-CAT (5 ng/well) was taken as 100%. Data are expressed as the mean ± S.E. of at least three independent experiments. * *p* < 0.05; *** *p* < 0.001, compared with wild-type (WT). §, *p* < 0.05 for the indicated comparison.

We previously reported that the expression of GATA2 is important for basal activation of rTRH(547)-CAT in CV1 cells that lack endogenous GATA2 expression [10]. We first compared GATA2 mutants with wild-type GATA2 for their ability to transactivate the *preproTRH* promoter. Interestingly, three mutations within ZF2 (T354M, R396Q, and R398W mutants) showed a marked reduction in the transcriptional ability of the *preproTRH* promoter. The transactivation potential of the R308P mutant was slightly lower than that of wild-type GATA2. We previously reported that GATA2 plays an essential role in the negative regulation of the *preproTRH* gene mediated by T_3_ [10]. As shown in Figure 2C, T_3_ treatment significantly reduced the transcriptional activity of rTRH(547)-CAT when wild-type *GATA2* and *S447R* were transfected. In contrast, negative regulation by T_3_ was not observed when the R308P mutant harboring mutation within ZF1 was transfected, although this may be explained by its decreased basal transcriptional activity. We did not evaluate the T_3_ responsivity of the three mutations within ZF2 because of their decreased basal transactivation potentials.

### 2.3. The GATA2 Mutants Have Reduced Transcriptional Activity on the TSHβ Promoter

**Figure 3:**
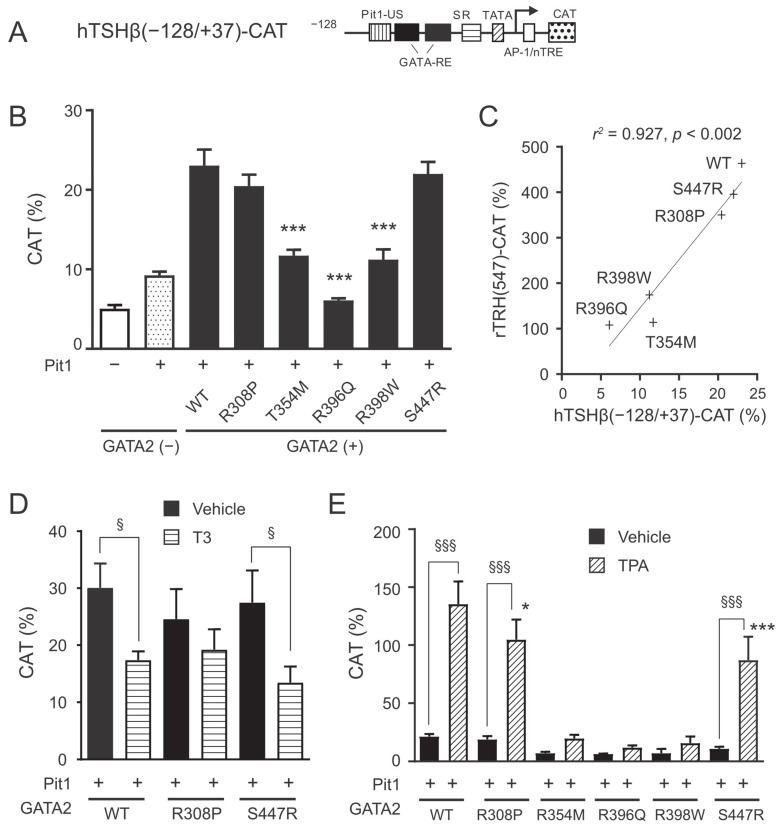
*GATA2* mutations impair basal *TSHβ* transcriptional activity and positive regulation by protein kinase C (PKC) pathway. (**A**) Schematic representation of hTSHβ(−128/+37)-CAT. GATA-RE, GATA responsive element; Pit1-US, Pit1-binding site upstream of GATA-REs; SR, suppressor region; AP-1, AP-1-binding site; nTRE, negative T_3_ responsive element; CAT, chloramphenicol acetyltransferase. (**B**) hTSHβ(−128/+37)-CAT (1.0 μg) was transfected into CV1 cells along with expression plasmids for various GATA2 mutants (0.2 μg), Pit1 (0.1 μg), TRβ2 (0.2 μg), and pCMV-β-galactosidase (0.2 μg). (**C**) Scattergram showing the relationship of hTSHβ(−128/+37)-CAT activity against rTRH(547)-CAT. The solid line is the regression line. (**D**) Transient transfection was conducted as described in (**B**). After 24 h of culture, the cells were treated with vehicle or 10 nM T_3_ for an additional 24 h. (**E**) hTSHβ(−128/+37)-CAT (1.0 μg) was transfected into CV1 cells along with expression plasmids for various GATA2 mutants (0.2 μg), Pit1 (0.1 μg), TRβ2 (0.2 μg), and pCH111-β-galactosidase (0.5 μg). After 24 h of culture, the cells were treated with 10 nM of the PKC activator tetradecanoylphorbol acetate (TPA) for an additional 24 h. CAT activity was normalized with β-galactosidase activity. CAT activity for pCMV-CAT (5 ng/well) was taken as 100%. Data are expressed as the mean ± S.E. of at least three independent experiments. * *p* < 0.05; *** *p* < 0.001, compared with wild type (WT). § *p* < 0.05; §§§ *p* < 0.001, for the indicated comparison.

We and others previously demonstrated that co-expression of GATA2 and Pit1 is important for the basal activation of hTSHβ(−128/+37)-CAT in CV1 cells [4,7,17]. In this study, GATA2 mutants were compared with the wild-type protein for their ability to transactivate the *TSHβ* promoter (Figure 3B). As observed in the *preproTRH* gene, three mutations within ZF2 (T354M, R396Q, and R398W mutants) resulted in a marked reduction in the transcriptional ability of the *TSHβ* promoter. For the R308P and the S447R mutants, the transactivation potential of the *TSHβ* promoter was slightly lower than that of the wild-type GATA2. The transcriptional ability of the *TSHβ* promoter correlated well with that of the *preproTRH* promoter (Pearson’s *r*^2^ value = 0.927, *p* < 0.002; Figure 3C). We previously reported that GATA2 is crucial for the negative regulation of *TSHβ* mediated by T_3_ [17]. T_3_ treatment significantly decreased the transcriptional activity of the *TSHβ* promoter when wild-type GATA2 and S447R were transfected. This negative regulation was not identified in R308P (Figure 3D), possibly due to reduced basal transcriptional activity. We previously reported that GATA2-dependent transcriptional activity of the *TSHβ* promoter is enhanced by liganded TRH receptors via the protein kinase C (PKC) pathway [29]. Treatment with the PKC activator tetradecanoylphorbol acetate (TPA) significantly enhanced the activity of the *TSHβ* promoter when *R308P* or *S447R* were transfected (Figure 3E). However, these two mutants showed a significant reduction in TPA-induced transactivation of the *TSHβ* promoter compared to the wild-type protein. *GATA2* mutation within ZF2 (*T354M, R396Q,* or *R398W*) demonstrated only minimal transcriptional activity of the *TSHβ* promoter under TPA treatment. 

### 2.4. DNA Binding Affinity of the GATA2 Mutants

**Figure 4:**
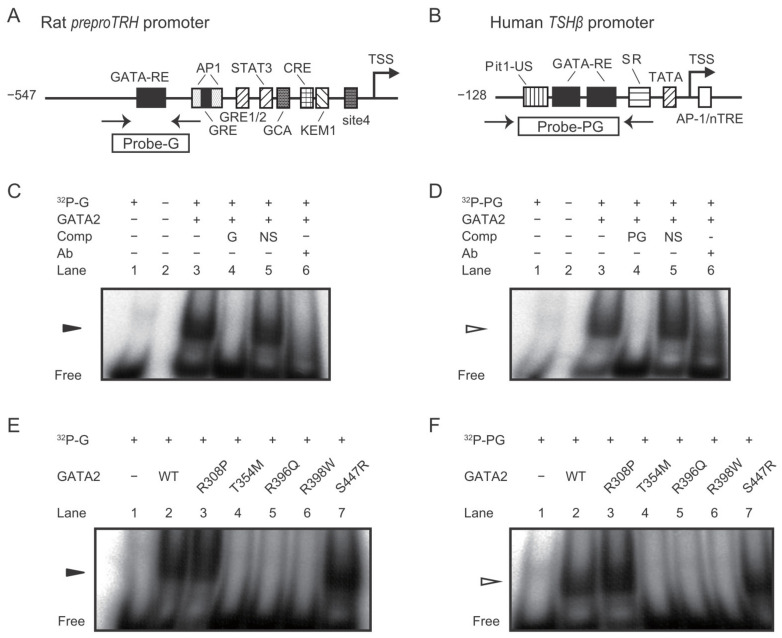
*GATA2* mutations impair their DNA binding properties on rat *preproTRH* and human *TSHβ* promoters. (**A**,**B**) Schematic representation of rat *preproTRH* and human *TSHβ* promoter. The positions of primers for the oligo DNAs for gel shift assay are indicated. GATA-RE, GATA responsive element; AP-1, AP-1-binding site; GRE, glucocorticoid responsive element; GRE1/2, glucocorticoid responsive element half-site; STAT, signal transducer and activator of transcription; CRE, cyclic AMP response element; GCA, GCA site; KEM1, KEM1 site; TSS, transcription start site; CAT, chloramphenicol acetyltransferase. Pit1-US; Pit1-binding site upstream of GATA-REs; SR, suppressor region; nTRE, negative T_3_ responsive element. (**C**,**D**) Gel shift assays demonstrated single bands (arrows) when ^32^P-labeled probe-G or probe-PG were incubated with nuclear extract of CV1 cells transfected with mouse *GATA2* expression plasmid. The specific band signal (lane 3) was abolished by a 50-fold amount of cold probe-G or probe-PG (lane 4) but not nonspecific double strand oligo DNA (NS; lane 5). These signals were supershifted when GATA2 protein was mixed with the anti-GATA2 antibody (ab) before incubation with ^32^P-labeled probes (lane 6). (**E**,**F**) Gel shift analysis with probe-G or probe-PG was conducted using nuclear extract of CV1 cells transfected with expression plasmids for various GATA2 mutants.

Next, we analyzed the DNA binding activity of wild-type and mutant GATA2 proteins on the conserved GATA responsive elements (GATA-REs) of the *preproTRH* and the *TSHβ* promoters using the gel shift assay. After CV1 cells were co-transfected with wild-type *GATA2* or *GATA2* mutants, the nuclear extracts were incubated with ^32^P-labeled DNA fragments harboring GATA-REs in the *preproTRH* or the *TSHβ* promoters (Figure 4A,B). In agreement with previous studies [10,29], the band corresponding to GATA2 was observed (lane 3; Figure 4C,D). These signals were abolished by the 50-fold specific competitor (lane 4) but not by unrelated DNA (lane 5) and were supershifted by a specific antibody against GATA2 (lane 6). Importantly, the three mutants harboring mutation within ZF2 (T354M, R396Q, and R398W) showed markedly reduced binding compared to wild-type GATA2 protein on *preproTRH* and *TSHβ* promoters (Figure 4E,F). In contrast to these three mutants, the DNA binding potential of R308P or S447R was equivalent to that of wild-type GATA2.

### 2.5. Protein Expression of the GATA2 Mutants

**Figure 5:**
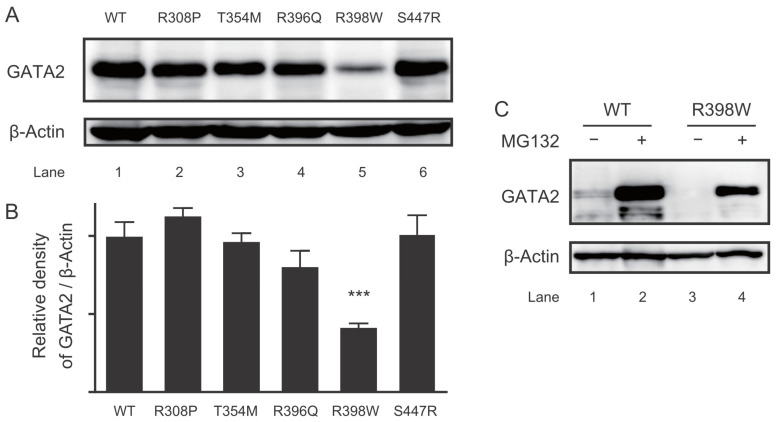
Decreased protein expression in GATA2 R398W via proteasome. (**A**) Representative image of Western blot with anti-FLAG antibody for the whole cell extract of CV1 cells transfected with FLAG-tagged expression plasmids for various GATA2 mutants. (**B**) Bar graph showing relative densitometric measurements for GATA2 quantification (*n* = 3). (**C**) Western blotting was conducted as described in (A) after CV1 cells were treated with vehicle or 10 μM MG132 for 24 h. *** *p* < 0.001, compared with wild type (WT).

We evaluated the protein expression of GATA2 mutants. Unexpectedly, a drastic reduction was observed in expression of the R398W mutant (Figure 5A). The protein expression of the remaining mutants was equivalent to that of wild-type GATA2 (Figure 5B). GATA2 was reported to be rapidly degraded by the ubiquitin–proteasome system [30,31]; thus, we examined the protein levels of R398W in the presence of MG132, a cell-permeable proteasome inhibitor. Interestingly, exposure to MG132 increased the protein expression of R398W (lane 4; Figure 5C). These findings suggest that enhanced ubiquitin–proteasome-dependent proteolysis caused a decreased protein expression of R398W. 

## 3. Discussion

Previous experimental and clinical studies demonstrated that *GATA2* mutations are associated with a wide range of human disorders [19,20,21,22,23,24,25,26,27,28]. In this context, we analyzed the effects of five different human *GATA2* mutations (*R308P, T354M, R396Q, R398W*, and *S447R*) on the HPT axis in vitro. Our findings revealed that these mutated GATA2 impaired the HPT axis through differential functional mechanisms. 

Three GATA2 mutants harboring mutation within ZF2 (T354M, R396Q, and R398W) exhibited markedly weak transcriptional activity and DNA binding potential compared to the wild-type protein toward the *preproTRH* and the *TSHβ* promoters (Figure 2, Figure 3 and Figure 4). Our findings are consistent with those of previous studies that focused on hematological disorders [22,25,27]. Chong et al. classified ZF2 mutants based on their DNA binding capabilities (class I, no significant reduction in binding; class II, reduced but detectable binding; and class III, little or no detectable binding) [27]. In their study, the T354M and the R398W mutants were classified as class II, whereas the R396Q mutant was classified as class III. The weakest basal transcriptional activity of R396Q on *preproTRH* and *TSHβ* promoters (Figure 2B and Figure 3B) in the present study may be explained by its lowest detectable binding capacity reported previously [27]. 

**Figure 6:**
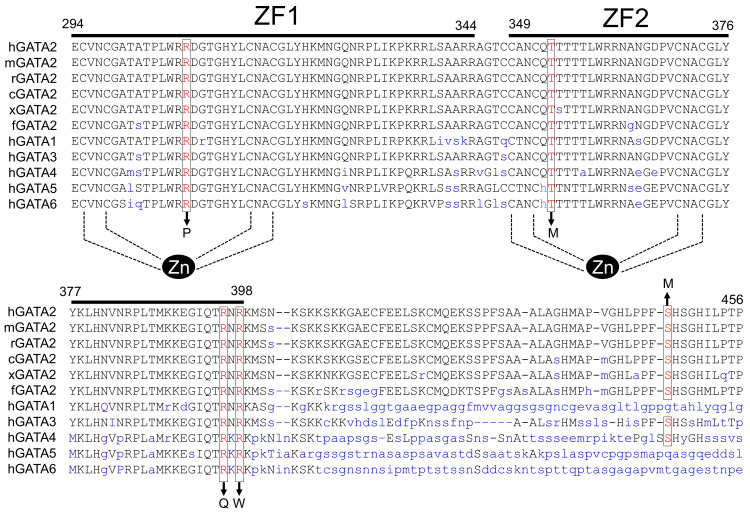
Amino acid sequences of the regions around the zinc (zn) fingers of the various GATA proteins. The amino acid sequences were retrieved from the National Center for Biotechnology Information database. Amino acid positions mutated in this study are indicated by boxes. The gene accession numbers for human GATA2 (hGATA2), mouse GATA2 (mGATA2), rat GATA2 (rGATA2), chicken (*Gallus gallus*) GATA2 (cGATA2), frog (*Xenopus laevis*) GATA2 (xGATA2), and fish (*Danio rerio*) GATA2 (fGATA2) are M68891, NM_008090, AY032734, NM_00100, XM_018256579, and NM_131233, respectively. The gene accession numbers for human GATA1, GATA3, GATA4, GATA5, and GATA6 are NM_002049, AY497006, AY740706, NM_080473, and NM_005257, respectively. ZF1, N-terminal zinc-finger domain; ZF2, C-terminal zinc finger domain.

The R398W mutant harboring mutation within ZF2 showed a marked reduction in protein expression (Figure 5A). Subsequent analysis with the proteasome inhibitor MG132 demonstrated increased protein expression of R398W (Figure 5B), suggesting that the mutant protein is prone to degradation through ubiquitin–proteasome-dependent proteolysis. The ubiquitin–proteasome pathway is the major degradation pathway for GATA2. All three ubiquitination target sites previously reported reside outside ZFs (amino acids 1–70, 153–256, and 412–480) [30,31]. The 398th amino acid is conserved among different species and isoforms (Figure 6). The effect of this mutant in other GATA families should be explored in future studies. It is also required to clarify the importance of the ubiquitin–proteasome system for the pathogenesis of GATA2 haploinsufficiency.

The S447R mutant harboring mutation within the C-terminal domain exhibited the same level of transactivation and DNA binding potential toward the *preproTRH* and the *TSHβ* promoters as the wild-type GATA2 protein (Figure 2B, Figure 3B, Figure 4E,F). However, the TPA-induced transactivation potential of the *TSHβ* gene was impaired in this mutant (Figure 3E). This might be attributed to post-translational modifications because the phosphorylation site resides in the C-terminal domain [32]. Hematological disorders were reported in at least three families with the S447R mutant [24,26]. Therefore, Haddox et al. hypothesized that missense mutations within the C-terminal domain might not simply abrogate GATA2 function but induce dominant-negative effects causing haploinsufficiency [33]; however, no functional analyses have been conducted to date. 

The R308P mutant harboring mutation within ZF1 showed intermediate characteristics between the ZF2 mutants and the C-terminal domain mutant. Basal transcriptional activity was slightly decreased in the *preproTRH* promoter (Figure 2B) but not in the *TSHβ* promoter (Figure 3B). DNA binding activities on these promoters were not impaired (Figure 4E,F), whereas the effect of TPA on *TSHβ* transactivation was lower than that of wild-type GATA2 (Figure 3E). It was reported that *GATA2* ZF1 mutations in CML are associated with a better prognosis than *GATA2* ZF2 mutations [34,35]. These findings suggest that GATA2 ZF1 mutants exert a limited influence on the HPT axis. 

The GATA2 ZF1 domain contributes to the stabilization of DNA binding and mediates its interaction with the transcriptional cofactor [36]. Greif et al. demonstrated that ZF1 mutants have a reduced capacity to cooperate with the transcription factor CEBPA to activate transcription [34]. Further studies are needed to clarify the contributions of transcriptional cofactors, including friend of GATA (FOG)-1 and 2, to GATA2-induced transactivation of *preproTRH* and *TSHβ* genes.

Previous in vivo and cell culture studies by us and others demonstrated that GATA2 plays an essential role in the HPT axis by regulating both the *preproTRH* and the *TSHβ* genes [4,5,6,7,10,17,18,29,37]. In this study, we further showed that naturally occuring *GATA2* mutations impaired GATA2 transcriptional activity toward the *preproTRH* and the *TSHβ* promoters in vitro. Because reduced pituitary TSH reserve and impaired thyrotropic stimulation by TRH are included in the pathophysiology of central hypothyroidism in humans [38], mutated GATA2 may contribute to develop this disorder.

Central hypothyroidism is characterized by defective thyroid hormone production caused by disturbed pituitary and hypothalamic functioning [38]. Inactivating mutations in genes encoding TSHβ, TRH receptor, and pituitary transcription factors are known causative mechanisms [39,40]. Recent studies using next-generation sequencing technology revealed numerous potential genes for heritable forms of central hypothyroidism [40,41]. For exmaple, Sugisawa et al. conducted the mutation screening study covering five genes (*IGSF1*, *IRS4*, *TBL1X*, *TRHR*, and *TSHB*) in Japanese patients with central congenital hypothyroidism and identified the *IGSF1* defect as the leading genetic cause in their patient cohort [42]. Genetic testing of *GATA2* mutations in patients with central hypothyroidism is an important issue that needs to be addressed further.

## 4. Materials and Methods

### 4.1. Plasmid Constructions

Since it was reported that the liganded TR artificially suppresses the firefly luciferase-based reporter gene [43], we employed CAT-based reporter genes in this present study. The rat *preproTRH* promoter, including nt. −547/+84, and the human *TSHβ* promoter, including nt. −128/+37, were fused to the CAT reporter gene to generate rTRH(547)-CAT and hTSHβ(−128/+37)-CAT, respectively, as described earlier [10,17]. Expression plasmids for human Pit1 (pCB^6^-hPit1) and human TRβ2 (pCMX-hTRβ2) were previously described [17]. Because *TRβ2* expression is specific to organs such as hypothalamus and pituitary, we employed the pCMX-hTRβ2 plasmid. The expression plasmid for FLAG-tagged human *GATA2* (pFLAG-CMV2-hGATA2) was gifted by Dr. Shinobu Tsuzuki (Aichi Medical University School of Medicine, Nagakute, Aichi, Japan). Mutant human *GATA2* expression plasmids carrying *R308P, T354M, R396Q, R398W*, and *S447R* were generated by site-directed mutagenesis using the QuikChange Site-Directed Mutagenesis kit (Agilent, Santa Clara, CA, USA). All mutated sequences were confirmed using Sanger sequencing.

### 4.2. Cell Culture and Transient Transfection

CV1 cells [44], a gift from Dr. Shunsuke Ishii (RIKEN Cluster for Pioneering Research, Tsukuba, Ibaraki, Japan), were grown in a monolayer culture at 37 °C in CO_2_/air (1:19) in Dulbecco’s modified Eagle’s medium containing 10% (*v*/*v*) fetal calf serum (FCS), penicillin G (100 units/mL), and streptomycin (100 μg/mL). CV-1 cells were trypsinized and plated in 12-well plates for 16 h before transient transfection. CV-1 cells at a density of 1 × 10^5^ cells per well were transfected using Lipofectamine reagent according to the manufacturer’s instructions (Promega, Madison, WI, USA). After cells were exposed to Lipofectamine reagent for 24 h, the medium was replaced with fresh media containing 5% (*v*/*v*) FCS depleted of thyroid hormone or media supplemented with T_3_ (0–10 nM). After incubation for an additional 24 h, the cells were harvested. CAT activity was normalized to the β-galactosidase activity. For each reporter assay, we performed transfection with pCMV-CAT, the magnitudes of which were adjusted to a value of 100%. Mycoplasma testing with a mycoplasma detection kit for conventional PCR, VenorGeM Classic (Minerva Biolabs, Berlin, Germany), was negative for CV1 cells used in this study.

### 4.3. Gel Shift Assay

Double-stranded oligo DNAs for G-probe (wild-type *preproTRH*, sense: 5′-agatgccacaagtccctatctcctttattttgctgc-3′ and antisense: 5′-gcagcaaaataaaggagatagggacttgtggcatct-3′) and PG-probe (wild-type *TSHβ*, sense: 5′-agtatgaattttcaatagatgcttttcagataagaaa-3′ and antisense: 5′-tttcttatctgaaaagcatctattgaaaattcatact-3′) were labeled with ^32^P-ATP using T4 polynucleotide kinase (Toyobo, Tokyo, Japan). CV-1 cells were transfected with pFLAG-CMV2 h*GATA2*, h*GATA2 R308P, T354M, R396Q, R398W*, or *S447R* (15 μg per 10 cm dish). After incubation for 24 h, the cells were harvested, and nuclear extracts were prepared as previously described [29]. ^32^P-labeled probes and 2 μg of nuclear extracts were incubated for 30 min on ice in 20 μL binding buffer containing 10 mM Tris-HCl (pH 7.6), 50 mM KCl, 0.05 mM EDTA, 2.5 mM MgCl_2_, 8.5% (*v*/*v*) glycerol, 1 mM dithiothreitol, 0.5 μg/mL poly (dI-dC), 0.1% TritonX-100, and 1 mg/mL nonfat dry milk. DNA–protein complexes were resolved by electrophoresis on a 5% (*w*/*v*) polyacrylamide gel at 150 V for 180 min at 4 °C. For the supershift assay, anti-GATA2 monoclonal antibody (a kind gift from Drs. Yasuharu Kanki and Tatsuhiko Kodama (The University of Tokyo and Perseus Proteomics Inc., Tokyo, Japan)) were added to the binding reaction mixture. The gel was dried, and labeled bands were visualized using an FLA-3000 autoradiography system (Fuji Film, Tokyo, Japan).

### 4.4. Western Blotting Analysis

Whole-cell extracts of CV-1 cells transfected with pFLAG-CMV2 h*GATA2*, h*GATA2 R308P*, *T354M, R396Q, R398W,* or *S447R* (5 μg per 6 cm dish) were fractionated by sodium dodecyl sulphate-polyacrylamide gel electrophoresis (SDS-PAGE) and subjected to Western blotting analysis with an anti-FLAG M2 antibody (F3165, Merck KGaA, Darmstadt, Germany). To assess the ubiquitin–proteasome-dependent degradation, whole-cell extracts of CV-1 cells transfected with pFLAG-CMV2 h*GATA2* or h*GATA2 R398W* in the presence or the absence of 1 μM MG132 (ChemScene, Monmouth Junction, NJ, USA) were fractionated by SDS-PAGE and subjected to Western blotting. The incubation time with MG132 was 6 h.

### 4.5. Statistics

All data are presented as mean ± standard error of the mean. Statistical significance of differences (*p* < 0.05) was examined by one-way analysis of variance followed by Fisher’s protected least significant difference using GraphPad PRISM v7.0 (GraphPad Software, Inc., San Diego, CA, USA).

## Data Availability

The data that support the findings of this study are available from the corresponding author upon reasonable request.

## Abbreviations

FCS	fetal calf serum
FOG	friend of GATA
GATA-RE	GATA responsive element
HPT axis	hypothalamus–pituitary–thyroid axis
PKC	protein kinase C
preproTRH	prepro-thyrotropin-releasing hormone
SDS-PAGE	sodium dodecyl sulphate–polyacrylamide gel electrophoresis
T_3_	triiodothyronine
TPA	tetradecanoylphorbol acetate
TR	thyroid hormone receptor
TRH	thyrotropin-releasing hormone
TSH	thyroid-stimulating hormone
ZF	zinc-finger domain
ZF1	N-terminal zinc-finger domain
ZF2	C-terminal zinc finger domain
